# Predictors and Factors Associated With Delayed Primary Cleft Surgery in Bangladesh

**DOI:** 10.7759/cureus.116577

**Published:** 2026-09-20

**Authors:** Tanmoy P Ghosh, Liman K Dhar, Diptee D Bithi, SM Ayub Hossain, Tamanna T Ahmed, Ishrat Jahan, Mohammad Rabiul K Khan

**Affiliations:** 1 Burn and Plastic Surgery, Mymensingh Medical College, Mymensingh, BGD; 2 Emergency Department, Upazilla Health Complex, Serajdikhan, Munshiganj, Dhaka, BGD; 3 Burn and Plastic Surgery, Khulna Specialized Hospital, Khulna, BGD; 4 Burn and Plastic Surgery, MH Samorita Hospital and Medical College, Dhaka, BGD; 5 Plastic and Reconstructive Surgery, National Institute of Burn and Plastic Surgery, Dhaka, BGD

**Keywords:** access to care, bangladesh, cleft lip, cleft palate, congenital heart disease, delayed presentation, delayed surgery, global surgery

## Abstract

Background

Delayed primary cleft surgery remains a major challenge in low- and middle-income countries (LMICs), where barriers to timely access coexist with medically necessary postponements. Understanding factors associated with delayed surgery and the relative contribution of presentation-related and medical reasons may help improve comprehensive cleft care. This study aimed to identify predictors of delayed primary cleft surgery, characterize the distribution of reasons for surgical delay, and evaluate factors associated with delayed presentation among patients treated in a comprehensive cleft care program in Bangladesh.

Methods

A retrospective cohort study was conducted at the National Institute of Burn and Plastic Surgery (NIBPS), Dhaka, Bangladesh. Medical records of patients undergoing primary cleft surgery between January 2016 and December 2020 were reviewed. Of 792 records screened, 424 met the eligibility criteria after exclusion of secondary or revision procedures, syndromic clefts, and incomplete records. Delayed primary surgery was operationally defined as primary cleft lip repair performed after 9 months of age or primary cleft palate repair performed after 18 months of age, according to predefined study criteria. Demographic and clinical variables were analyzed using univariate and multivariable binary logistic regression to identify factors associated with delayed primary surgery. A secondary multivariable analysis evaluated factors associated with operationally defined delayed presentation. Six prespecified predictor variables were included in the multivariable models.

Results

Among 424 patients, 132 (31.1%) underwent delayed primary cleft surgery. Operationally defined delayed presentation was the most frequently identified reason for delayed surgery, accounting for 70 (53.0%) delayed cases, followed by congenital heart disease (16.7%), anaemia (12.1%), and other causes (18.2%). In multivariable analysis, urban residence was independently associated with lower odds of delayed primary surgery compared with rural residence (adjusted OR 0.642, 95% CI 0.418-0.988; p=0.044), whereas congenital heart disease was associated with higher odds of delayed primary surgery (adjusted OR 2.720, 95% CI 1.424-5.197; p=0.002). For delayed presentation, congenital heart disease was independently associated with lower odds of delayed presentation (adjusted OR 0.205, 95% CI 0.048-0.876; p=0.032). Residence, gender, cleft phenotype, other congenital anomalies, and anaemia were not significantly associated with delayed presentation.

Conclusion

Nearly one-third of patients experienced delayed primary cleft surgery, with delayed presentation being the most frequent documented contributor. Rural residence and congenital heart disease were independently associated with delayed primary surgery, whereas residence was not independently associated with delayed presentation. Congenital heart disease was independently associated with lower odds of delayed presentation, a finding that should be interpreted cautiously. These findings highlight the need to distinguish potentially preventable access-related delays from medically indicated postponements when planning strategies to improve timely cleft care.

## Introduction

Cleft lip with or without cleft palate (CL±P) is among the most common congenital craniofacial anomalies, affecting approximately one in every 700 live births worldwide, although substantial geographic and ethnic variations exist [1,2]. Children born with cleft deformities frequently experience feeding difficulties, recurrent otitis media, hearing impairment, speech disorders, dental abnormalities, facial growth disturbances, and psychosocial challenges that adversely affect health-related quality of life. Consequently, comprehensive cleft care requires coordinated multidisciplinary management extending from infancy into adulthood [1-3].

Primary surgical repair is the cornerstone of cleft management, and the timing of repair varies among cleft teams and treatment protocols worldwide. Primary cleft lip repair may be performed from the neonatal period through approximately six months of age or later [4], while the timing of primary palatoplasty also varies according to institutional protocols and treatment priorities. Early palatal repair has generally been favored to support speech development, whereas late repair may allow better maxillary growth [5]. Evidence from retrospective studies has also suggested differences in midfacial growth according to the timing of palatal repair [6]. These variations in recommended timing, institutional practice, and referral patterns may contribute to differences in the age at which patients undergo primary cleft surgery. In resource-constrained settings, where access to specialised cleft services may be influenced by referral pathways and geographical barriers, understanding the factors associated with surgery outside the intended treatment window is particularly important. Therefore, the present study evaluated the frequency, reasons, and predictors of delayed primary cleft surgery using prespecified age thresholds for lip and palatal repair within a comprehensive cleft-care program in Bangladesh. 

Despite considerable advances in multidisciplinary cleft management, delayed primary surgery remains common in many low- and middle-income countries (LMICs). The reasons are multifactorial and include delayed presentation to specialised cleft centres, limited parental awareness, inadequate referral pathways, geographic barriers, socioeconomic disadvantage, transportation difficulties, shortages of specialised surgical services, and medical comorbidities requiring preoperative optimisation such as congenital heart disease (CHD), anaemia, malnutrition, or respiratory infections [7-10]. Although medically indicated postponements are often unavoidable, delays resulting from late presentation represent potentially preventable barriers to timely surgical care.

Bangladesh has substantially expanded cleft care through collaborations between government institutions, academic hospitals, and international charitable organisations, particularly Smile Train, leading to improved access to reconstructive surgery [11]. Nevertheless, delayed primary cleft surgery remains frequently encountered in routine clinical practice. Published studies from Bangladesh have primarily focused on epidemiology, nutritional status, surgical techniques, and associated congenital anomalies, whereas relatively little attention has been paid to determinants of delayed surgery or to the relative contribution of delayed presentation compared with medical causes of postponement. Understanding these factors is essential for strengthening referral systems, improving community awareness, optimising multidisciplinary perioperative care, and ensuring equitable access to comprehensive cleft services.

Accordingly, this study aimed to identify independent predictors of delayed primary cleft surgery among patients managed within a comprehensive cleft care program in Bangladesh. In addition, we sought to characterise the principal causes of delayed surgery and to perform a secondary exploratory analysis of factors associated with delayed presentation, the most common potentially preventable contributor to delayed surgical treatment. These findings may help clinicians, policymakers, and cleft care organisations develop targeted interventions to reduce avoidable delays and improve access to timely cleft surgery.

## Materials and methods

Study design and study population

This retrospective cohort study was conducted at the National Institute of Burn and Plastic Surgery (NIBPS), Dhaka, Bangladesh, the country's only comprehensive cleft care centre. The institute provides multidisciplinary cleft services through collaboration with a charitable organisation, CLEFT-UK [1], including plastic surgery, orthodontics, speech therapy, paediatric care, nutritional support, and anaesthesia services.

Consecutive medical records of 792 patients who underwent cleft surgery at NIBPS between January 2016 and December 2020 were reviewed. Medical records were screened for eligibility according to predefined inclusion and exclusion criteria.

Patients were eligible if they had non-syndromic cleft lip (CL), cleft palate (CP), or cleft lip and palate (CLP) and underwent primary cleft surgery during the study period. Only records containing complete demographic, clinical, and perioperative information were included.

Records were excluded if they involved secondary or revision cleft procedures (n = 249), syndromic clefts (n = 22), or contained incomplete medical records (n = 97). Following eligibility assessment, 424 patients met the study criteria and were included in the final analysis (Figure 1). The study was reported in accordance with the Strengthening the Reporting of Observational Studies in Epidemiology (STROBE) guidelines [12].

**Figure 1:**
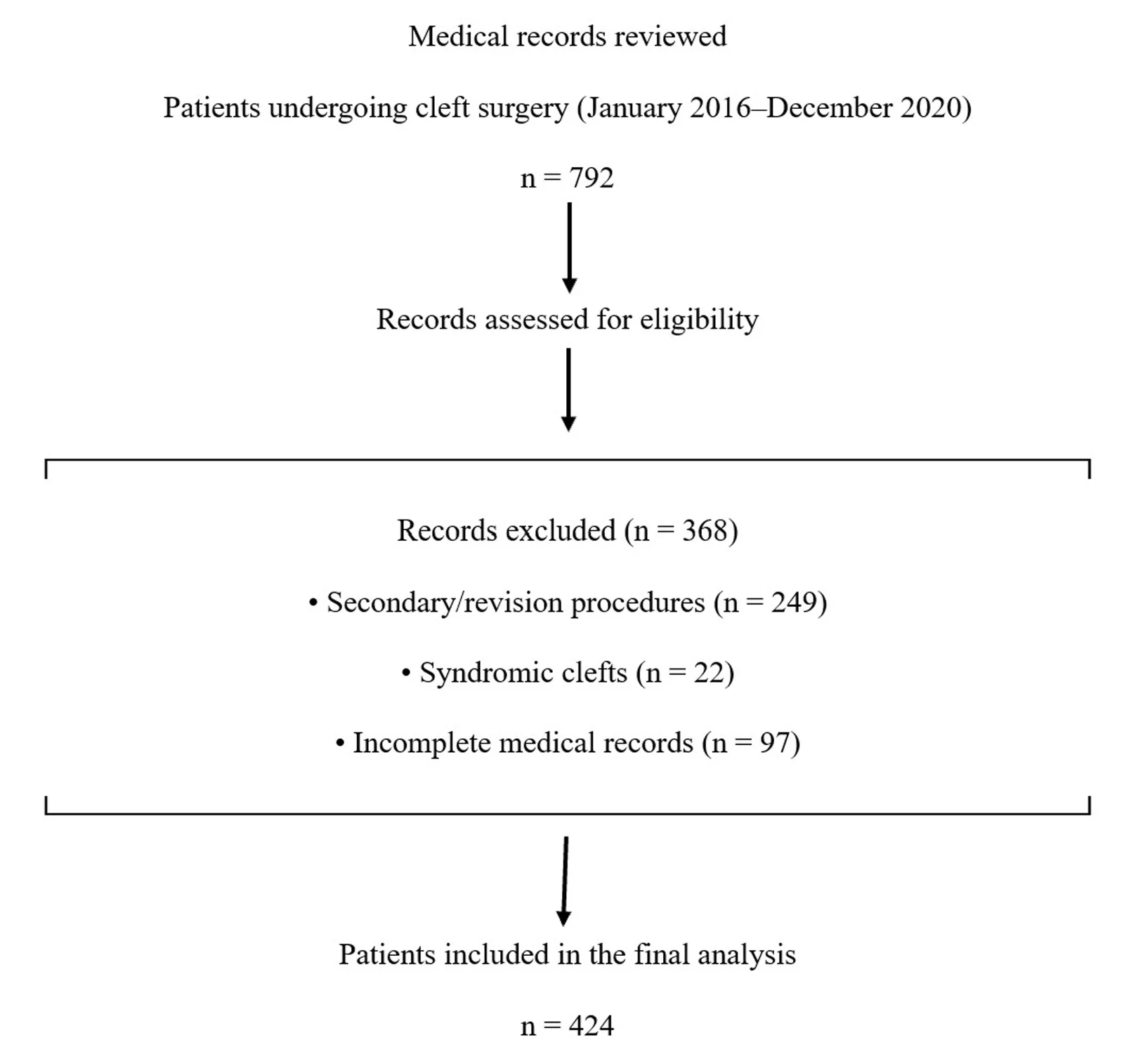
Flow diagram illustrating the selection of study participants. Of 792 medical records of patients undergoing cleft surgery reviewed between January 2016 and December 2020, 368 were excluded because they represented secondary or revision procedures, syndromic clefts, or had incomplete medical records. The remaining 424 patients fulfilled the eligibility criteria and were included in the final analysis.

Data collection

Data were retrospectively extracted from hospital operative registers and medical records using a standardised data collection form. Variables collected included age at first presentation to the cleft service, age at primary surgery, gender, residence (urban/rural), cleft phenotype, CHD, anaemia, other congenital anomalies, delayed primary surgery, and the recorded reason for surgical delay. Delayed presentation was subsequently operationally classified from the recorded age at first presentation according to the predefined study criteria. Consanguinity was recorded when available but excluded from multivariable analyses due to substantial missing data and limited relevance to the primary study objective. Data extraction was performed by the first author using a standardised data collection form.

Statistical analysis

Data were analysed using IBM SPSS Statistics version 26.0 (IBM Corp., Armonk, NY, USA). Continuous variables are presented as mean ± standard deviation (SD), while categorical variables are expressed as frequencies and percentages. Associations between categorical variables were initially explored using the Chi-square test or Fisher's exact test, as appropriate. Variables considered clinically relevant based on previous literature and biological plausibility, including gender, residential location, cleft phenotype, CHD, anemia, and other congenital anomalies, were initially evaluated using univariate binary logistic regression to estimate crude odds ratios (ORs) with 95% confidence intervals (CIs).

For the multivariable analyses, the six prespecified variables were simultaneously entered into a binary logistic regression model using the Enter method, irrespective of their statistical significance in univariable analysis. This approach was used to obtain adjusted estimates while accounting for clinically important potential confounding. Cleft lip and palate was used as the reference category for cleft phenotype, while rural residence, male gender, absence of CHD, absence of anaemia, and absence of other congenital anomalies were used as the respective reference categories.

Multicollinearity among the independent variables was assessed using tolerance, variance inflation factor (VIF), and collinearity diagnostics. No evidence of significant multicollinearity was identified (tolerance 0.967-0.997; VIF 1.003-1.034). Model calibration was evaluated using the Hosmer-Lemeshow goodness-of-fit test, while explanatory performance was assessed using the Nagelkerke R² statistic. For the delayed-surgery model, the Hosmer-Lemeshow test showed no evidence of poor model fit (χ²=3.576, df=8, p=0.893). For the delayed-presentation model, the corresponding result was χ²=2.252, df=7, p=0.945.

There were 132 delayed-surgery events among 424 patients. The six prespecified predictor variables corresponded to 22 events per predictor variable (EPV). Because cleft phenotype comprised three categories and was represented by two indicator variables, the final model contained seven estimated regression parameters. Thus, with the 132 delayed-surgery events, the event-to-predictor-variable ratio was 22, while the parameter-based event-per-parameter ratio was approximately 18.9. All statistical tests were two-sided, and a p-value <0.05 was considered statistically significant.

Operational definitions

The timing of primary cleft lip repair varies among cleft teams worldwide, ranging from neonatal repair to repair at 6 months of age or later [4]. Similarly, the optimal timing for primary palatoplasty varies among cleft teams. Palate repair before 12 months of age has been considered favourable for speech development, whereas late repair has been associated with less restriction of maxillary growth [5]. In one retrospective study, palatal repair at 18-20 or 21-24 months was associated with better midfacial growth than repair at 9-11 months [6]. Considering the variation in published recommendations, institutional practices, and referral patterns, for the purposes of this study, delayed primary cleft surgery was defined a priori as primary cleft lip repair performed after 9 months of age and/or primary cleft palate repair performed after 18 months of age. These thresholds were prespecified before analysis and applied consistently across patients with cleft lip only (CL), cleft palate only (CP), and cleft lip and palate (CLP). For patients with CLP or those requiring multiple primary procedures, the age at the first primary cleft operation was used to determine whether surgery was delayed.

The documented causes of delayed surgery were categorised as: delayed presentation, CHD, anaemia, and other causes.

Delayed presentation was operationally defined as first presentation to the multidisciplinary cleft service after the predefined age threshold used to classify delayed primary surgery (>9 months for cleft lip and >18 months for cleft palate), in the absence of documented medical conditions necessitating postponement. This variable was retrospectively derived from the recorded age at first presentation and was intended to identify late entry into the cleft-care pathway rather than delay occurring after presentation to the specialist service. The category "other causes" included temporary conditions such as acute respiratory tract infection, urinary tract infection, pyrexia of unknown origin, and postponement or delay due to operating room scheduling or hospital workload.

Anaemia was defined as any documented haemoglobin (Hb) level <11.0 g/dL, considered the standard definition by WHO [7].

CHD is identified here by reviewing echocardiographic and paediatric cardiologist opinion-related documents by the cardiologist of the multidisciplinary team of NIBPS. Atrial septal defect (ASD), ventricular septal defect (VSD), patent ductus arteriosus (PDA), and significant tricuspid regurgitation (TR) cases were included. Trivial tricuspid regurgitation (TR), mitral regurgitation (MR), and patent foramen ovale (PFO) cases were not included in CHD definitions.

Outcome measures

The primary outcome was delayed primary cleft surgery (Yes/No).

The secondary outcomes were the causes of delayed surgery and factors associated with delayed presentation, including the most common cause of surgical delay.

## Results

A total of 424 patients who underwent primary cleft surgery between 2016 and 2020 were included in the analysis. Of these, 222 (52.4%) were male, and 202 (47.6%) were female. Most patients were from rural areas (n=234, 55.2%), while 190 (44.8%) resided in urban areas. The most common cleft phenotype was cleft lip and palate (50.9%), followed by isolated cleft palate (36.6%) and isolated cleft lip (12.5%). Consanguinity was documented in 75 (17.7%) patients, absent in 84 (19.8%), and unknown in 265 (62.5%) (Table 1). Mean age at presentation was 20.8 ± 51.4 months. The median age at first presentation was nine months (IQR, 5-15 months; range, 2-720 months), while the median age at primary surgery was 10 months (IQR, 6-15 months; range, 3-720 months). The broad age range at first presentation and primary surgery (2-720 months) reflected inclusion of both paediatric and adult patients. CHD was present in 44 (10.4%) patients, anaemia in 213 (50.2%), and other congenital anomalies in 21 (5.0%).

**Table 1:** Baseline characteristics of the study population (N = 424)

Variable	Category	n	%
Sex	Male	222	52.4
	Female	202	47.6
Residence	Urban	190	44.8
	Rural	234	55.2
Cleft phenotype	Cleft lip	53	12.5
	Cleft palate	155	36.6
	Cleft lip and palate	216	50.9
Consanguinity	Yes	75	17.7
	No	84	19.8
	Unknown	265	62.5
Delayed surgery	No	292	68.9
	Yes	132	31.1

Delayed primary surgery occurred in 132 patients (31.1%), whereas 292 (68.9%) underwent surgery within the recommended timeframe (Table 2). The most common contributor to delayed surgery was delayed presentation, accounting for 70 patients (53.0%) of all delayed cases (Figure 2). Other major causes found were CHD (16.7%) and anaemia (12.1%). This indicates that delayed presentation is the predominant factor contributing to delayed surgery. The distribution of causes of delay differed significantly (p<0.001) (Table 2).

**Table 2:** Causes of delay in primary cleft surgery (n=424) *Fisher's exact test CHD=congenital heart disease

Parameter		Frequency	Ratio (%) among all patients (n=424)	Ratio (%) among delayed surgeries (n=132)	p-value
No Delay		292	68.9	-	
Delay		132	31.1	100	
Causes of delay	CHD	22	5.2	16.7	<0.001*
	Anaemia	16	3.8	12.1	
	Delayed presentation	70	16.5	53.0	
	Others	24	5.7	18.2	

**Figure 2:**
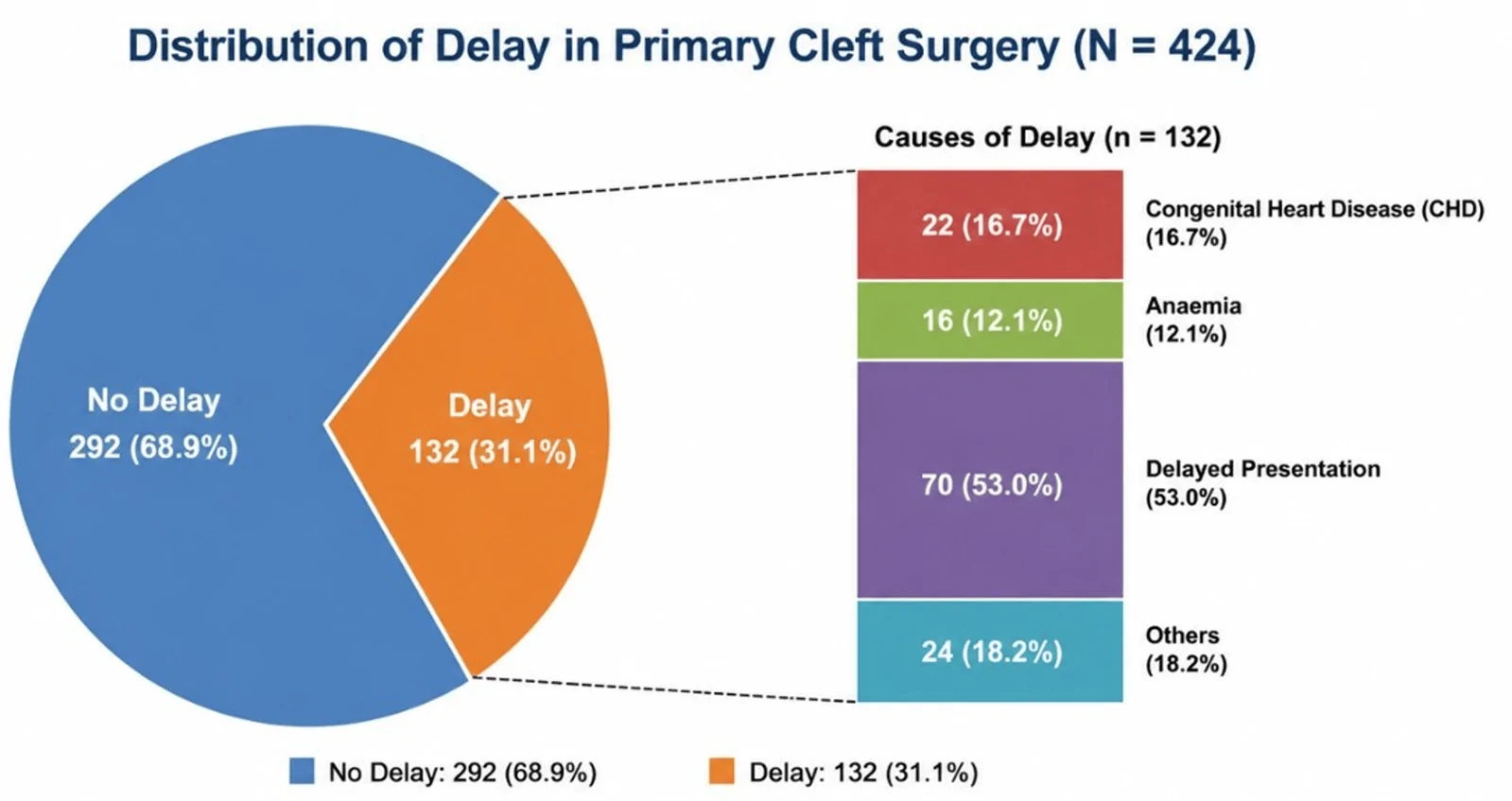
Distribution of delays in primary cleft surgery (n=424)

In univariate logistic regression analysis (Table 3), urban residence was significantly associated with lower odds of delayed primary cleft surgery compared with rural residence (crude OR 0.63, 95% CI 0.42-0.96; p=0.033). Gender was not significantly associated with delayed surgery (crude OR 1.10, 95% CI 0.73-1.66; p=0.657). Using cleft lip and palate as the reference phenotype, neither cleft lip only nor cleft palate only was significantly associated with delayed surgery (crude OR 0.84, 95% CI 0.43-1.63; p=0.608 and crude OR 0.96, 95% CI 0.61-1.50; p=0.842, respectively); the overall association between cleft phenotype and delayed surgery was also not significant (χ²=0.270, df=2, p=0.874). Other congenital anomalies were not significantly associated with delayed surgery (crude OR 1.39, 95% CI 0.56-3.43; p=0.481). Similarly, anaemia was not significantly associated with delayed surgery (crude OR 1.40, 95% CI 0.93-2.12; p=0.107). In contrast, CHD was significantly associated with higher crude odds of delayed surgery (crude OR 2.72, 95% CI 1.45-5.12; p=0.002).

**Table 3:** Univariate logistic regression analysis for factors associated with delayed primary cleft surgery (N=424) OR=odds ratio; CI=confidence interval; CHD=congenital heart disease

Predictor	Crude OR	95% CI	p-value
Residence			
Rural (reference)	1.00		
Urban	0.63	0.42-0.96	0.033
Gender			
Male (reference)	1.00		
Female	1.10	0.73-1.66	0.657
Cleft phenotype			
Cleft lip and palate (reference)	1.00		
Cleft lip only	0.84	0.43-1.63	0.608
Cleft palate only	0.96	0.61-1.50	0.842
Other congenital anomalies			
No (reference)	1.00		
Yes	1.39	0.56-3.43	0.481
Anaemia			
No (reference)	1.00		
Yes	1.40	0.93-2.12	0.107
CHD			
No (reference)	1.00		
Yes	2.72	1.45-5.12	0.002

Variables considered clinically relevant were entered into a multivariable logistic regression model to identify independent predictors of delayed primary cleft surgery (Table 4). In multivariable logistic regression analysis, residence and CHD were independently associated with delayed primary cleft surgery. Compared with rural patients, those residing in urban areas had significantly lower adjusted odds of delayed surgery (aOR 0.642, 95% CI 0.418-0.988; p=0.044). In contrast, patients with CHD had significantly higher adjusted odds of delayed surgery compared with those without CHD (aOR 2.720, 95% CI 1.424-5.197; p=0.002). Gender, cleft phenotype, anaemia, and other congenital anomalies were not significantly associated with delayed surgery after adjustment. The overall model was statistically significant (χ2= 17.109, df=7; p=0.017), although explanatory power was limited (Nagelkerke R2= 0.056). The Hosmer-Lemeshow test showed no evidence of poor model fit (χ²=3.576, df=8, p=0.893).

**Table 4:** Multivariable logistic regression analysis for factors associated with delayed primary cleft surgery OR=odds ratio; CI=confidence interval; CHD=congenital heart disease

Predictor	Adjusted OR	95% CI	p-value
Residence			
Rural (reference)	1.000		
Urban	0.642	0.418-0.988	0.044
Gender			
Male (reference)	1.000		
Female	1.126	0.732-1.733	0.589
Cleft phenotype			
Cleft lip and palate (reference)	1.000		
Cleft lip only	0.849	0.427-1.690	0.642
Cleft palate only	0.881	0.552-1.406	0.596
Other congenital anomalies			
No (reference)	1.000		
Yes	1.446	0.573-3.648	0.435
Anaemia			
No (reference)	1.000		
Yes	1.344	0.877-2.059	0.175
CHD			
No (reference)	1.000		
Yes	2.720	1.424-5.197	0.002

Because delayed presentation was the leading factor contributing to delayed primary cleft surgery, a secondary multivariable logistic regression analysis was performed to identify factors associated with delayed presentation (Table 5).

**Table 5:** Multivariable logistic regression analysis for factors associated with delayed presentation OR=odds ratio; CI=confidence interval; CHD=congenital heart disease

Predictor	Adjusted OR	95% CI	p-value
Residence			
Rural (reference)	1.000		
Urban	0.652	0.381-1.115	0.118
Gender			
Male (reference)	1.000		
Female	0.905	0.529-1.546	0.714
Cleft phenotype			
Cleft lip and palate (reference)	1.000		
Cleft lip only	1.235	0.540-2.824	0.617
Cleft palate only	1.162	0.654-2.062	0.609
Other congenital anomalies			
No (reference)	1.000		
Yes	2.156	0.792-5.870	0.133
Anaemia			
No (reference)	1.000		
Yes	1.110	0.650-1.862	0.723
CHD			
No (reference)	1.000		
Yes	0.205	0.048-0.876	0.032

In this multivariable logistic regression analysis, CHD was independently associated with delayed presentation. Patients with CHD had significantly lower odds of delayed presentation compared with those without CHD (aOR 0.205, 95% CI 0.048-0.876; p=0.032). Residence, gender, cleft phenotype, other congenital anomalies, and anaemia were not significantly associated with delayed presentation. The overall association between cleft phenotype and delayed presentation was not statistically significant (χ2=0.400, df=2; p=0.819). The overall multivariable model for delayed presentation was not statistically significant (χ2= 11.549, df=7; p=0.116) and had limited explanatory power (Nagelkerke R2=0.045). The Hosmer-Lemeshow test showed no evidence of poor model fit (χ²=2.252, df=7, p=0.945).

## Discussion

The present study evaluated predictors of delayed primary cleft surgery and the documented reasons for treatment delay in Bangladesh's national referral cleft-care setting. To our knowledge, this represents one of the largest single-centre retrospective cohorts evaluating predictors of delayed primary cleft surgery in Bangladesh. Approximately one-third (31.1%) of patients underwent delayed primary surgery. More importantly, this study distinguished delays associated with documented medical conditions requiring postponement from delays associated with operationally defined delayed presentation. Although this classification was based on predefined study criteria, it provides a clinically useful framework for describing the relative contributions of medical and presentation-related factors to the delay in primary cleft surgery.

The principal finding of this study was that delayed presentation accounted for more than half (53.0%) of all delayed primary surgeries, making it the single largest contributor to treatment delay within the present study framework. In contrast, medically indicated postponements due to CHD (16.7%) and anaemia (12.1%) together accounted for less than one-third of delayed procedures. These findings suggest that the greatest opportunity for reducing delayed cleft surgery lies not in modifying perioperative medical management but in improving timely access to specialised cleft care. Similar observations have been reported in recent systematic reviews, which identified delayed diagnosis, poor caregiver awareness, transportation difficulties, financial hardship, fragmented referral systems, and limited access to specialist services as the principal barriers to timely cleft treatment in LMICs. Massenburg et al. also emphasised that health-system barriers rather than surgical capacity frequently determine the timing of cleft repair in resource-constrained settings [2,3].

Medical conditions such as congenital heart disease and significant anaemia often necessitate postponement of elective surgery to minimise anaesthetic and perioperative risk [7]. Such delays represent appropriate clinical decision-making rather than deficiencies in service delivery. Conversely, operationally defined delayed presentation reflects late entry into the cleft-care pathway in the absence of documented medical contraindications. Although this may indicate potentially remediable barriers to timely access, the retrospective design and absence of detailed socioeconomic and health-system variables preclude definitive conclusions regarding the underlying mechanisms responsible for delayed presentation. Recognising this distinction has important implications for clinical audit, quality improvement, and health policy because interventions to reduce delayed presentation differ fundamentally from those required to optimise medically complex patients [8,9].

Residential location emerged as an independent predictor of delayed primary cleft surgery, but not of delayed presentation after multivariable adjustment. Compared with patients from rural areas, those residing in urban areas had significantly lower adjusted odds of delayed surgery (aOR 0.642, 95% CI 0.418-0.988; p=0.044). This finding suggests that geographical or healthcare-access factors may influence whether patients undergo primary cleft surgery within the predefined age window. However, because residence was not independently associated with delayed presentation (aOR 0.652, 95% CI 0.381-1.115; p=0.118), the present data do not establish that rural residence independently determines late entry into cleft care. Socioeconomic variables, travel distance, transportation costs, referral pathways, and access to specialist services were not available in the retrospective dataset; therefore, the mechanisms underlying the observed association cannot be determined. Rural residence may nevertheless serve as a marker of unmeasured structural factors. Future prospective studies incorporating socioeconomic and health-system variables are needed to clarify these relationships. Previous reports from LMICs demonstrate that long travel distances, transportation costs, delayed referral from peripheral health facilities, socioeconomic disadvantage, and limited availability of specialist services disproportionately affect rural populations [10-15].

CHD accounted for approximately one-sixth of delayed surgeries in the present series and was significantly associated with delayed primary surgery in univariate analysis (crude OR 2.72, 95% CI 1.45-5.12; p=0.002). This association remained significant after multivariable adjustment, with patients with CHD having higher odds of delayed primary surgery than those without CHD (aOR 2.720, 95% CI 1.424-5.197; p=0.002). This finding is clinically plausible because patients with CHD may require cardiological evaluation, optimisation of cardiovascular status, and additional anaesthetic assessment before elective cleft repair. Such postponement may represent appropriate patient-safety considerations rather than deficiencies in cleft-care delivery. Likewise, anaemia represented another important medical indication for postponement until physiological optimisation had been achieved. These findings support current multidisciplinary cleft care protocols that prioritise patient safety before elective reconstruction and should not be interpreted as deficiencies in clinical care. Recent reviews of perioperative management of children with congenital heart disease similarly recommend postponing elective procedures until appropriate cardiovascular stabilisation has been achieved [16-19].

Neither gender, cleft phenotype, nor associated congenital anomalies independently predicted delayed surgery after multivariable adjustment. These findings suggest that, within the variables examined, residential location may be more relevant to whether surgery occurs within the predefined age window than demographic characteristics or cleft phenotype. Comparable findings have been reported in several contemporary studies, where socioeconomic and healthcare accessibility variables were stronger determinants of treatment delay than patient demographics or cleft morphology [3,14,15].

The multivariable logistic regression analysis (Table 5) identified congenital heart disease as the only independent predictor of delayed presentation. Patients with CHD had significantly lower odds of delayed presentation than those without CHD (aOR 0.205, 95% CI 0.048-0.876; p=0.032). This inverse association should, however, be interpreted cautiously because of the operational definition of delayed presentation used in this study. Delayed presentation was defined as presentation beyond the predefined age threshold in the absence of documented medical conditions necessitating postponement; therefore, patients whose late surgery was attributable to significant CHD were classified under medically indicated postponement rather than delayed presentation. Consequently, the observed inverse association between CHD and delayed presentation may substantially reflect this classification structure rather than a true protective effect of CHD against late presentation. The overall model was not statistically significant (χ2= 11.549, df=7; p=0.116) and had limited explanatory power (Nagelkerke R^2^=0.045 ), suggesting that important determinants of delayed presentation were not captured by the variables available in the retrospective dataset. Residence, gender, cleft phenotype, other congenital anomalies, and anaemia were not independently associated with delayed presentation. 

The present study represents one of the largest hospital-based studies from Bangladesh examining delayed primary cleft surgery within a standardised multidisciplinary treatment program. The study employed multivariable regression analyses to identify independent predictors of delayed surgery and, importantly, differentiated medical postponements from potentially avoidable delays. This distinction provides clinically actionable information for improving cleft care delivery and has rarely been emphasised in previous studies.

Several limitations should also be acknowledged. First, the retrospective design limited analyses to variables routinely documented in medical records and precluded assessment of potentially important socioeconomic and health-system determinants of healthcare access. In addition, 97 records were excluded because they lacked sufficient information for analysis, introducing the possibility of selection bias. Second, the study was conducted at a single national referral centre, and the patient population may therefore be influenced by referral patterns and differential access to specialised cleft services. This referral and access bias may limit generalizability of the findings to patients receiving care in other regions or healthcare settings. Third, although model calibration was assessed using the Hosmer-Lemeshow test, discrimination statistics such as the area under the receiver operating characteristic curve (AUC) were not available; therefore, the overall discriminatory performance of the models could not be fully assessed. Fourth, the documented reasons for delayed surgery were retrospectively abstracted from clinical records and may have been incompletely recorded or misclassified, particularly when more than one factor contributed to postponement. Interobserver agreement for data abstraction was not assessed because data extraction was performed retrospectively by a single investigator. Finally, because delayed presentation was operationally defined using the recorded age at first presentation and the same predefined age thresholds used to classify delayed primary surgery, some conceptual overlap between delayed presentation and the primary outcome is unavoidable. Accordingly, delayed presentation should be interpreted as an operational indicator of late entry into the cleft-care pathway rather than a completely independent explanatory variable. Future prospective studies using the interval between first presentation and surgery, together with prospectively documented reasons for delay, would better distinguish delays occurring before referral from those occurring after entry into specialist care.

In addition, the explanatory power of the two multivariable models was limited. The delayed-surgery model had a Nagelkerke R²=0.056, whereas the delayed-presentation model had a Nagelkerke R²=0.045. This suggests that important determinants of delayed surgery and, particularly, delayed presentation were not captured by the demographic and clinical variables available in the retrospective dataset. Future prospective studies incorporating socioeconomic, educational, transportation, referral, and health-system variables are therefore warranted.

Overall, the findings indicate that delayed primary cleft surgery is influenced by both potentially modifiable access-related factors and clinically necessary postponements. Rural residence was independently associated with delayed primary surgery, whereas it was not independently associated with delayed presentation after adjustment. These findings highlight the importance of strengthening referral pathways and equitable access to specialised cleft services, particularly for rural populations, while maintaining appropriate perioperative optimisation for patients with medical comorbidities. Future prospective studies incorporating socioeconomic, transportation, referral, and health-system variables are warranted to identify the mechanisms underlying delayed presentation and to guide targeted interventions.

## Conclusions

Nearly one-third of patients undergoing primary cleft surgery experienced delayed treatment. Delayed presentation was the most frequently documented reason for delayed surgery, while CHD and anaemia were important medical reasons for postponement. Rural residence and CHD were independently associated with delayed primary surgery, whereas residence was not independently associated with delayed presentation. The findings support efforts to improve timely referral and equitable access to specialised cleft care while preserving appropriate perioperative optimisation for medically complex patients. Strengthening referral pathways, early case identification, community awareness, and equitable access to multidisciplinary cleft services may help reduce avoidable delays in primary cleft surgery. These findings provide a preliminary framework for clinical audit and may inform future quality-improvement initiatives and cleft-care policy in Bangladesh.
